# NKR-P1A/CD161: A Multifunctional Immune Receptor at the Crossroads of Antitumor Immunity

**DOI:** 10.3390/biom16091302

**Published:** 2026-09-09

**Authors:** Chiara Rosa Maria Uras, Martina Taglieri, Linda Di Gregorio, Alessandro Poggi

**Affiliations:** 1 Molecular Oncology and Angiogenesis Unit, Azienda Ospedaliera Metropolitana Liguria AOM-IRCCS Ospedale Policlinico San Martino, 16132 Genoa, Italy; chiararosamaria.uras@aomliguria.it (C.R.M.U.); martina.taglieri@aomliguria.it (M.T.); linda.digregorio@aomliguria.it (L.D.G.); 2 Dipartimento di Medicina Sperimentale (DIMES), University of Genoa, 16132 Genoa, Italy

**Keywords:** tumor immunology, innate cells, MAIT, NK cells, immune regulation

## Abstract

The immune response of our body, whether it is directed to infective agents, allergens, or tumors, should support our recovery but can also be dangerous if dysregulated. There are molecular systems that have the role of tuning the immune response, such as activating and inhibitory receptors and co-stimulatory molecules. Among this plethora of receptors, immune-checkpoint receptors (ICRs), as well as natural killer receptors (NKRs), expressed on NK and/or T cells can up- or downregulate the function of activating and inhibitory receptors during immune cell crosstalk. Among NKRs, NKRP1A/CD161 has shown to be a promising target molecule for immunotherapy. This receptor showed inhibiting or co-stimulatory effects depending on the cell type analyzed as well as the analytical procedures, reagents used, and the experimental context. The aim of the following review will be to explore the structural and functional features of this receptor on immune cells, its relevance in the tumor microenvironment and its targeting as an immunotherapeutic tool in cancers.

## 1. Introduction

The expression of inhibitory surface molecules on immune cells is essential to properly regulate the degree and time of the innate and adaptive arms of the immune system [1,2,3] The dysregulation of inhibiting signals in immune cells can lead to the clinical appearance of autoimmunity, while the expression on tumor cells of ligands for the inhibiting molecule of anti-tumor effector cell-mediated activity can favor the growth and dissemination of tumor cells [4,5,6]. On the other hand, the regulation of immune response is necessary to maintain the homeostasis of healthy tissues [2,7,8]. It is conceivable that there is a fine balance between activating and inhibiting signals to avoid any self-aggression but maintain an appropriate response to microorganisms and stressed signals [9,10,11,12]. Nowadays, antagonism with monoclonal antibody (mAb) or small molecules of immune inhibitory receptors is a key therapeutic tool to reawaken the immune response to trigger the response to tumor cells. Paradigmatic examples are represented by anti-PD1/PDL1 and/or anti-CTLA4 mAbs used for the treatment of several hematological and solid tumors in association with chemotherapeutics or tyrosine kinase inhibitors [13,14,15,16,17]. Although there is evidence that the regulatory network is far too limited to PD1 and CTLA4 checkpoint molecules, it is composed of multiple receptors that may interact with cell surface ligands on tumor cells to downregulate the immune response [18,19,20,21,22]. This complex scenario may be the reason for the unsatisfactory or limited clinical results reported in the literature exclusively targeting PD1 and CTLA4 [23,24,25,26,27,28]. Many functional molecules have been identified in natural killer (NK) cells as receptors (NKRs) involved in either activation or inhibition of NK-cell-mediated anti-tumor effect [29,30,31,32,33,34]. Among the multitude of NKRs, some of them show superimposable molecular features in the outer cell membrane portion with the presence of different molecular intracellular domains, such as what is reported for killer Ig-like inhibitory receptor (KIR). Indeed, inhibitory KIR bearing tyrosine-based inhibition motif (ITIM) and activating KIR associated with adaptor proteins bearing tyrosine-based activation motif (ITAM) after engagement with the same HLA-I allele can trigger either inhibition or activation of immune cell response [35,36,37,38,39,40]. On the other hand, human natural killer receptor protein 1A (NKRP1A/CD161) does not show a molecular structure or an association with either ITIM or ITAM components, but it may show an inhibitory functional behavior on various immune cells [41,42,43]. On the other hand, human NKRP1A/CD161 engagement by specific mAbs can trigger the activation of Ca^2+^/calmodulin-dependent kinase II (CAMKII) [44] or phosphatidylinositol 3-kinase (PI3K)-dependent activation of the protein kinase B (PKB/Akt) and extracellular signal-regulated kinase (ERK) biochemical pathways [41]. These findings would be in contrast to each other, as the engagement of NKRP1A/CD161 may lead to the overall accepted role of immune inhibition together with positive signals for immune response. The murine and rat counterparts will be briefly considered to highlight the evidence that different NKRP1 molecules recognize different ligands and deliver activating or inhibiting signals in anti-tumor effector cells, at least in rodents. Furthermore, NKRP1A/CD161 is a molecule expressed by immune cells associated with infectious, allergic and autoimmune diseases, besides the role played in immune response to tumors. Herein, we will specifically analyze some of the reported roles of human NKRP1A/CD161 affecting immune response to tumors, trying to unify the different points of view on its function, and targeting immunotherapy.

## 2. NKRP1A/CD161: Molecular Structure

NKRP1A/CD161 is an integral membrane protein of type II, coded by the Killer Cell Lectin-Like Receptor B1 (*KLRB1*) gene, which was cloned by the group of Lanier et al. in 1994 [45]. In the original description, this transmembrane protein was considered an inhibitory receptor regulating tumor-target-directed cell cytotoxicity [45]. Apparently, NKRP1A/CD161 binds neither calcium ions nor carbohydrate ligands, differently from the classical C-type lectin receptors (CLRs), such as dendritic-cell-specific intercellular adhesion molecule-3-grabbing non-integrin (DC-SIGN), C-type lectin domain family 4 member A (CLEC4A), and mannose-binding lectin (MBL) [46,47]. Instead, NKRP1A interacts with the CLEC2D (or osteoclast inhibitory lectin molecules, OCILs) protein encoded by lectin-like transcript-1 (*LLT1*) [46,48] genetically linked to the NKRP1-coding *KLR* gene. This interaction system is important for non-MHC “missing-self” and “induced-self” recognition. The first one consists of the identification and destruction of target cells lacking or MHC I complex or with low expression. The second one is the identification and elimination of “abnormal” cells based on their surface self-antigens [46,48,49,50]. NKRP1 receptors were described first in mice and rats and then in humans, but NKRP1A (CD161, *KLRB1* gene) is the only human orthologue described since 1994 [45]. It should be noted that the rat NKRP1 family members designated as NKRP1 A, B, C, D, F, and G show either activating or inhibiting/regulating functions, while human NKRP1A/CD161 was originally described as an inhibiting surface receptor. Nevertheless, based on structural and functional homology to NKRP1, other human C-type lectin-like receptor (CLR)–ligand pairs such as NKp65-KACL (KLRF2-CLEC2A) [50] and NKp80-AICL (KLRF1-CLEC2B) [51] have been proposed as the activating counterparts of human NKRP1 [50,52]. The coding gene for NKRP1A is localized on chromosome 12, which contains the NK gene complex (*NKC*), as well as the genes for CD69 and CD94 [53]. The complex is composed of genes coding for NKR-PB1 and other genes coding for CLRs, proteins that are structural homologs and interact with NKRP1A/CD161 [54]. NKRP1A/CD161 is made up of three regions: a 158-aminoacid extracellular domain with motifs typical of C-type lectins, with several C residues and N-linked glycosylation sites; a 29-aminoacid transmembrane domain; and a 38-aminoacid cytoplasmic domain [45,46].

## 3. NKRP1A/CD161 Expression Across Immune Cell Subsets

NK cells represent the first lymphocyte subset in which NKRP1A/CD161 was detected. During the workshops of the Leukocyte Typing VI conference on white cell differentiation antigens held in Kobe in 1996 [55], it was observed that three different anti-NKRP1A antibodies, namely DX12, HP-3G10, and 191B8, reacted specifically with NIH-3T3 human NKRP1A transfected cells, but not with untransfected NIH-3T3 cells. In addition, DX12 and HP-3G10 displayed a similar MFI, while 191B8 mAb showed an 8-fold stronger MFI. This would suggest that the original three anti-NKRP1A mAbs can recognize different epitopes of the target molecule, or that they link with a different affinity for the corresponding antigen. The analysis of several leukocyte subsets indicated that the reactivity of the three mAbs was similar on polyclonal NK cell populations, while it was markedly different on NK cell clones expressing either CD158a or CD158b killer Ig-like inhibitory receptors for HLA class I alleles. These findings should be considered with attention in evaluating the functional behavior of the NKRP1A/CD161 molecule in each research study. NK cells can express NKRP1A/CD161, and its engagement reduces the surface expression of CD107a, a marker of degranulation, and the release of IFN-γ, a cytokine inducing cell cytotoxicity [56,57]. Regarding innate immune cells, NKRP1A/CD161 is also expressed on monocytes and monocyte-derived dendritic cells, while in the adaptive immune system NKRP1A/CD161 is expressed on different subsets of peripheral αβ T lymphocytes, either CD4^+^ or CD8^+^, or γδ T cells [58]. As regards T cells, NKRP1A is expressed only on memory T lymphocytes, and it is upregulated in different T cell subsets present in peripheral blood or localized tissue, such as mucosal-associated invariant T (MAIT) cells [59,60,61,62,63,64,65,66,67,68]. Of note, NKRP1A/CD161 expression can be induced with the stimulation of NK cells and T cells with phorbol 12-myristate 13-acetate (PMA) and IL-2. It can also be detected in circulating monocytes and B cells [69,70]. The expression of NKRP1A on putative antigen-presenting cells (APCs) has also been observed in rats, especially on a fraction of peripheral Lew rat monocytes [71]. In detail, the size of NKRP1A^+^ monocytes was significantly increased after the administration of IFN-γ. The NKRP1A^+^ L-selectin^+^ CD43^low^ CD4^−^ monocytes were also present in the peripheral blood of rats during immune reactions [71]. This finding would suggest that NKRP1A^+^ monocyte subpopulations may have a role in processes leading to immune response. Table 1 summarizes NKRP1A expression and some homologous molecules in immune cells across rodents and humans.

The NKRP1A/CD161 molecule is expressed by all IL-17-producing lymphocytes, and it is induced by the RAR-related orphan receptor C transcription factor (RORc, RORγt in humans) [85], which is the master regulator of IL-17 cell differentiation. Furthermore, the progenitor of Th17 cells resides within the CD4^+^CD161^+^ T cell [86], and NKRP1A/CD161 is highly expressed on peripheral blood of allergic rhinitis patients compared to healthy controls [87]. These cells potentially can produce IL-17, a cytokine of inflammatory disorders also involved in the preservation of barrier integrity and mucosal immunity [88,89,90]. However, NKRP1A/CD161 can be expressed by peripheral T cells producing other cytokines, such as IFN-γ and TNF-α, in addition to IL-17 [91]. In addition, the NKRP1A^+^ peripheral CD3^+^ T cell frequency has been analyzed in asthma patients in stable condition and during asthma attack [92]. NKRP1A/CD161^+^ T cells increased during the acute phase of asthma. This finding further supports the relevance of NKRP1A/CD161 as a molecule characterizing immune cells undergoing antigen-mediated activation.

### NKRP1A/CD161 Expression on Tumor-Infiltrating Lymphocytes and Peripheral Blood of Tumor-Bearing Patients

The expression of NKRP1A/CD161 in cancers has been studied extensively to determine whether this molecule can identify lymphocytes involved in a putative immune response against the tumor. Furthermore, the degree and the frequency of the expression of this molecule have been studied in several types of cancers to determine its association with the prognosis of the disease. Based on the findings described in the previous chapter, the expression of NKRP1A/CD161 would be associated with both immune activation and the classical immunoregulatory cytokine IL-17. This would suggest both anti-tumor and pro-tumor effects associated with the presence of NKRP1A/CD161^+^ immune cells. In a group of patients suffering from breast, ovarian, pancreas, colon, stomach, and lung cancers or pleural effusion and ascites of metastasis of these cancers, the expression of NKRP1A/CD161 was analyzed both in peripheral blood (PB) and in tumor-infiltrating lymphocytes (TILs) [93]. Importantly, the proportion of CD4^+^ T cells expressing CD161 was higher in the PBMCs of cancer patients as well as in TILs. The increase in the amount of CD4^+^CD161^+^ T cells was associated with cancer progression, with a significant difference between early tumor stages I and II versus late tumor stages (III and IV). CD4^+^CD161^+^ and CD4^+^CD161^−^ cells differently expressed CD95, CD45RO, and CD45RA. Importantly, CD45RA expression decreased while CD45RO expression increased in cancer patients compared to healthy donors [86]. This kind of expression was more marked on mononuclear cells from metastasis. These findings would indicate that NKRP1A is expressed on activated T cells both in peripheral blood and in TILs [94,95]. More interestingly, there are some conflicting reports on the prognostic relevance associated with the expression of the NKRP1A/CD161 antigen on some subsets of TILs [86,87,88,89]. Indeed, human papillomavirus (HPV)-driven oropharyngeal carcinoma (OPSCC) CD161 has been identified as a potential marker to characterize CD8^+^ cytotoxic T lymphocyte subsets in HPV-positive OPSCC with a divergent evolutionary trajectory. These cells, present both in peripheral blood and in TILs, showed robust anti-tumor immunity and an inflamed phenotype expressing higher IFN-γ, TNF-α, and granzyme B compared to their CD161-negative counterparts. These CD8^+^CD161^+^ T cells displayed a reversible state instead of terminal dysfunction, and they were reinvigorated with immunotherapy. Indeed, they were enriched with immune checkpoints such as PD1, CD39, and Tim3. Of note, the infiltration with CD161^+^ CTLs is associated with better treatment response and prolonged survival. A survival benefit was found in OPSCC patients with high infiltration of active CD4^+^CD161^+^ T cells as well [87]. Indeed, both central and effector memory CD4^+^ T cells express CD161, but only effector memory CD161^+^ T cells showed an association with improved survival in OPSCC. They expressed elevated levels of the transcriptional transactivator *SOX-4*, which is involved in enhancing the triggering of T cells through TCR. Furthermore, these cells appeared to rapidly respond to suboptimal antigen stimulation, suggesting that CD161, as well as *SOX4*, is involved in CD3-mediated triggering [96,97,98]. Importantly, the expression of CD161 was downregulated by TGF-β on CD4^+^ T cells, while the same cytokine upregulated the expression of PD1 and CD39. This finding indicates that some immune checkpoint molecules display opposite behavior regarding their expression compared to CD161. On the other hand, NKRP1A/CD161 expression was not associated with a better outcome in gliomas [99,100]. In fact, NKRP1A/CD161 was enriched in isocitrate dehydrogenase (IDH) wild-type and high-grade gliomas and in the mesenchymal subtypes. More relevantly, the NKRP1A/CD161 antigen could be considered an independent prognostic factor for the overall survival of patients with gliomas. These findings would suggest that CD161 is closely related to the molecular and pathological features of some glioma subtypes, and it plays a role in the immune dysfunction present in these tumors. In addition, the pan-cancer bioinformatic analysis performed on a large cohort of tumors [101] identified two major groups of genes with either favorable or negative prognostic value, in which the *KLRB1* gene coding for CD161 belongs to an mRNA signature associated with a better prognosis, in contrast to the *FOXM1* gene, which is linked to a worse prognosis [101]. Higher *KLRB1* expression was linked to a lower survival rate only in renal carcinoma, while all the others showed a better prognosis. It should be noted that this analysis comprised gliomas as well. The conflicting results reported for gliomas can be explained considering the different cohorts of patients analyzed. Furthermore, the lack of analysis in the same report for the protein expression of NKRP1A/CD161 together with bioinformatic data of mRNA of the *KLRB1* gene can provide conflicting results. Thus, the expression of NKRP1A/CD161 should be confirmed using complementary methodological approaches such as flow cytometry, immunohistochemistry, and mRNA analysis.

However, the biological significance of the expression of CD161 on TILs should be analyzed within the specific TME and the contemporary expression of sets of genes and corresponding proteins that may influence the progression and evolution of cancers. Furthermore, studies on breast cancer have shown that CD161/NKRP1A expression is downregulated in breast carcinomas and that it is correlated with poor overall survival and relapse-free survival [102,103]. In hepatocellular carcinoma (HCC), single-cell profiling from about 17,000 cells from 18 primary or relapsed HCCs showed that in recurrent tumors there was an increase in TIL CD8^+^ lymphocytes overexpressing CD161. These cells displayed a low cytotoxic state with innate-like features, and the enrichment in these cells was associated with a poor prognosis [104]. However, the analysis of TCGA data indicated a strong correlation between *KLRB1* expression and immune T cell activation. Indeed, single-cell data on tissue resident NK and T cells displayed activation markers. Furthermore, the downregulation of *KLRB1* expression on PB T and NK lymphocytes compared to healthy donors correlated with poor prognosis [105]. These two reports are in part conflicting, although the analysis in the first instance was on CD8^+^ T cells, while in the second report the analysis was on the whole T and NK cell populations. Regardless, *KLRB1* may represent a marker associated with prognosis in HCC. To define a positive or negative correlation with the HCC outcome, a larger cohort of patients is necessary to avoid bias in the analysis resulting from small sample cohorts or unselected subsets of immune cells. It is also noted that the function of NKRP1A/CD161 may depend on the expression in the tumor and on the various components of the TME of the known natural ligand and the molecular mechanisms involved in their expression.

## 4. The Interaction Between NKRP1A/CD161 and CLEC2D: Structural and Functional Implications

The main ligand of NKRP1A/CD161 is the C-type lectin domain family 2 member D (CLEC2D), encoded by the gene lectin-like transcript 1 (*LLT1*) in human and named OCIL or C-type lectin related (Clrb, Clec2d) in mice and rats [106]. The structural characterization by Kamishikiryo et al. in 2011 of NKRP1A/CD161 has shown that the interaction with CLEC2D depends on membrane-distal β sheet and loop regions of both molecules, allowing a molecularly robust receptor–ligand binding [107].

### 4.1. Cellular Expression of CLEC2D on Immune Cells

The *LLT1* gene is primarily expressed in hematopoietic cells, and exon skipping generates some splicing variants, such as variant 1, coding for the form of CLEC2D expressed at the cell surface (isoform 1) [106]. Two other transcript variants encode for transmembrane protein isoforms 2 and 4, which are intracellular and whose function is still not clear [105]. CLEC2D is not expressed in humans on resting leukocytes, but it is positive on germinal center B cells and early plasmablasts, while in mice it is constitutively expressed on leukocytes, and on some non-hematopoietic cells [106,107,108,109]. The expression of *LLT1* is inducible in various cell types. In particular, in mice its expression can be stimulated in TLR-activated plasmacytoid dendritic cells (pDCs) and B cells, as well as TCR-activated T cells and IL-2-activated NK cells [108,109]. In humans, it is inducible in B cells, osteoblasts, and chondrocytes, and on tumor cells of prostate cancer, non-small cell lung cancer (NSCLC), and triple-negative breast cancer (TNBC) [110,111,112]. *LLT1* transcripts have been detected in some hematopoietic cells at low levels, such as T, B, and NK cells, but not monocytes, and the protein expression was almost absent on these cells in resting conditions [111]. Overall, the reported expression on resting immune cells of CLEC2D may be conflicting, as the analyses have been performed with different procedures and reagents. On the other hand, the reported upregulation on activated immune cells is consistent among the reports [106,107,108,109,110,111,112,113,114]. In vitro stimulation with PHA or anti-CD3 plate coating was found to induce the exposure of CLEC2D on the T cell surface, after heavy glycosylation [111], in association with CD69 presence, promoting cell activation. The induction of CLEC2D cell surface expression was also observed on activated invariant NKT cells, as well as on CD4+ T cells secreting IFN-γ-, IL-4-, IL-17, and IL-10. The cross-linking of CD16 can also induce CLEC2D surface expression in NK cells [109]. In addition, studies on mature dendritic cells revealed that bacterial stimuli such as LPS can induce CLEC2D exposure on their surface through the triggering of TLR4-mediated intracellular pathway. Also, inflammatory stimuli, such as IFN-γ, could increase CLEC2D expression on the cell surface of mature dendritic cells, differently from cytokines such as IL-12, IL-4, IL-17, and IL-10 [112], inducing STAT1 transcriptional activation. Also, through the in vitro infection of HIV and EBV viruses, an increase in the surface exposure of CLEC2D on B cells was observed, confirming the relationship between infection and CD161 receptor expression [111]. Overall, the surface expression of CLEC2D can be induced by TLR signaling and inflammasome-TNF-IL-1 axes, which can amplify its presence in infected, inflamed, and tumor microenvironments (TMEs) [99,113,114]. Furthermore, CLEC2D was detected by immunohistochemistry on germinal center B lymphocytes of tonsils, the spleen, Peyer’s patches, and lymph nodes. In addition, this molecule is expressed on plasmablasts with a suggestive appropriate phenotype showing CD45^low^, CD20-, CD19^low^, CD79a^−/low^, pan Ig^+^, MUM-1^+/−^, CD38^+^, Ki67^+/−^, and CD138^−^ reactivity. On the other hand, follicular dendritic cells (FDCs), neutrophils, macrophages, and plasmablasts matured into plasma cells were negative for CLEC2D [102]. Table 2 summarizes the cellular distribution of CLEC2D and clec2d in humans and rodents, respectively. Within tumors, CLEC2D can be expressed on tumor cells as well as leukocytes and other components of the TME [113,114,115,116,117]. Its expression, together with that of its ligand NKRP1A/CD161, can have a role both in tissue homeostasis and in response to inflammation, infection and tumorigenesis [117,118,119].

### 4.2. Expression of CLEC2D on Hematologic Malignancies and Solid Tumors

A. Calderon and colleagues analyzed the expression of *LLT1* on hematological malignancies, showing that *LLT1* RNA and the CLEC2D protein are highly expressed in B-cell lymphoma, T-cell lymphomas, and lymphocytic and myelogenous leukemias [120]. Indeed, analysis of RNA-seq data from a large panel of cancer cell lines from both hematological malignancies (208 cell lines) and other tumors (1131 cell lines) revealed that CLEC2D was well expressed on B cell lymphomas such as diffuse large B cell lymphomas, mantle cell lymphomas, Hodgkin lymphomas, acute lymphocytic leukemia and chronic lymphocytic leukemias (CLLs), in addition to multiple myeloma and AML and CML. This wide expression was confirmed also at the cell surface with either anti-CLEC2D antibody or chimeric Fc-NKRP1A/CD161 molecules. In addition, *LLT1* is expressed on different solid tumors, although with a strong heterogeneity among the donors and at a lower degree compared to hematological malignancies [118,120]. *LLT1* expression was analyzed using TCGA transcriptome data, and flow cytometry was conducted to confirm the bioinformatic data [118]. The high expression of *LLT1*, compared to that of adjacent non-tumor healthy tissue, was found in several cancers, such as BRCA, CHOL, ESCA, GBM, HNSC, KIRC, KIRP, LIHC, LUAD, STAD, SARC, and PCPG. The pan-cancer methylation of *LLT1* revealed that its expression was negatively associated with methylation levels. In addition, the copy-number variation (CNV) of *LLT1* was heterozygous amplification or deletion, with minor types being homozygous amplification or deletion. In certain cancers such as KIRC, high *LLT1* expression was associated with poor prognosis. In COAD and KICH, despite the low expression, *LLT1* was associated with a worse prognosis. On the other hand, the expression of *LLT1* was associated with a favorable prognosis in BRCA, LUAD, HNSC, BLCA, and CESC. Analysis of *LLT1* expression was performed in association with the TME infiltration of anti-tumor effector cells such as CD3^+^CD8^+^ T cells or CD4^+^ regulatory cells. In LIHC, LUAD, PAAD, KIRC, PRAD, LUSC, SKCM, THCA, BRCA, KIRP, STAD, ESCA, and THYM, the expression of *LLT1* was positively associated with the presence of anti-tumor effector T cells, while in in LIHC, PAAD, CHOL, and THYM, it was positively associated with the presence of Treg cells and *LLT1* expression but negatively associated with CD4^+^ naïve T cells. Furthermore, TIGIT, LAG3, CD274 (PDL1), HAVCR2 (TIM-3), IL4, IL10, IDO1, ARG1, ICOS, CD39, CCL17, FOXP3, and CD33 immunoregulatory genes were positively associated with LLT1. Accordingly, a negative association was found with LAMP1 (CD107a), IL-1B, A2M, PTEN, HLA-E, ULBP1, LKB-1, BAD, and SLAMF9 proinflammatory and oncosuppressor genes. Finally, the CRI iAtlas portal was interrogated to define the expression of LLT1 transcripts in patients who did not respond to immune checkpoint therapies across multiple solid cancers. The tumors analyzed were STAD, BLCA, GBM, KIRC, and SKCM. It should be noted that a considerable proportion of patients suffering from the above-mentioned tumors showed a high expression of LLT1, associated with a lack of response to ICI blockers. The SKCM nonresponders to the anti-PD1 nivolumab showed that TIGIT, LAG3, CD274 (PDL1), IDO1, ICOS, ENTPD1, IL10, and FOXP3 genes were associated with *LLT1*. These results would indicate that *LLT1* may function in association with other ICI and other immunoregulatory genes to create a potent immune suppressive TME [118]. However, the described analysis coming from the TCGA database includes primary and metastatic tumors that can show different biological features, and the absence of complete staging information for all cancer may give biased results and interpretations. Single-cell RNA-seq analysis can be a good approach to further characterize the phenotype and the functional state of either tumor or immune cells. Single-cell RNA-seq analysis represents a further step in defining the feature of tumor cells expressing *LLT1* [113]. Indeed, CLEC2D is localized with glial fibrillary–acidic protein (GFAP)-positive astrocytes but not with Iba-1 as a microglial marker. CLEC2D was co-expressed with the NLR family caspase activation and recruitment domain (CARD)-containing 4 (NLRC4) inflammasome [115]. Indeed, the expression of the NKRP1A/CD161 ligand can be associated with the expression of pro-inflammatory molecules such as the NLRC4 inflammasome component in tumor-associated astrocytes during glioblastoma, correlating with poor prognosis [115]. Likewise, the NLRC4 inflammasome was regulated in glioma cells by Tim3/Gal9 [115]. Thus, different surface-regulating molecules such as the CLEC2D receptor CD161/NKRP1A and Tim3/Gal9 are associated with the poor prognosis of GBM. Overall, there is some evidence that the interaction between CD161/NKRP1A and CLEC2D may play a key role in the regulation of anti-tumor immune response and the outcome of some hematologic and solid tumors.

## 5. NKRP1A/CD161-CLEC2D Interaction Signaling: Activation or Inhibition of Effector Cell Function?

Surface membrane receptors expressed on lymphocytes could be subdivided into two main groups that can deliver either an activating or an inhibiting signal [22,27,121]. The specific mAb is the practical tool that, by recognizing a specific molecule, may mimic the interaction between the ligand and the receptor. The functional consequences of the use of anti-NKRP1A/CD161 mAb added to a cellular system may reveal the role of this receptor. In addition, to study the features of NKRP1A-mediated signaling, some valuable information can come from the use of soluble ligands such as soluble CLEC2D or Fc Ig chimeric molecules. Herein, we will report the experimental evidence regarding the function of NKRP1A/CD161 using these tools and discuss the reason for the dual behavior of this receptor.

### 5.1. Inhibiting Effects of NKRP1A/CD161 Targeting

Based on these premises, in NK cells NKRP1A/CD161 has been considered an inhibitory receptor [43,45,122] with weak affinity with the CLEC2D molecule. The CLEC2D homodimer can interact with NKRP1A/CD161 in a bivalent way. Indeed, one dimer of CLEC2D can interact with two NKRP1A dimers; the link is on different portions of the NKRP1A dimeric molecules. Indeed, NKRP1A forms homodimers in an unexpected arrangement to enable CLEC2D binding in two modes, bridging two CLEC2D molecules. These interaction clusters are suggestive of an inhibitory immune synapse. The ligation of CLEC2D with NKRP1A leads to the clustering of NKRP1A and the use of soluble CLEC2D, affecting the killing of K562 cells mediated by NK cells and supporting the notion that NKRP1A is an inhibitory receptor [122]. Further, this notion is supported by the data of the first report on the function of NKRP1A in NK cells [45]. Indeed, the cross-linking of NKRP1A/CD161 with a specific mAb can inhibit the killing of the FcγR+ murine mastocytoma cell line P815 but not that of NK-sensitive target cells [45]. Accordingly, it has been shown that during killing mediated by IL-2-activated NK or IL-2-activated CD2^−^ CD3^−^ thymocytes of the P815 murine cells, the human myelomonocytic cell lines U937 and HL60 and the adenocarcinoma cell line SI.2 were inhibited using specific anti-NKRP1A mAb. This inhibitory effect was not detected for the killing of K562 cells [43]. It is of interest that in the same experimental system, antibodies to lymphocyte-function-associated antigen (LFA)1 can show a strong inhibitory effect to all the cell lines used, including K562 cells. It is well known that LFA1, expressed on cytolytic effector cells and recognizing its counter receptor, ICAM1, is involved in the binding between effector and target cells. This binding is impaired using anti-LFA1-specific mAb [123,124,125]. On this basis and based on the similarities between the effects of anti-NKRP1A/CD161 mAb and anti-LFA1 mAb, it is possible that the NKRP1A/CD161 receptor may function as an adhesion molecule like LFA-1 [123,124]. The effect of the addition of an anti-NKRP1A/CD161 mAb should impair the interaction between NKRP1A/CD161 and its counter ligand CLEC2D; thus, the inhibitory effect detected could be not related to an inhibitory signal transduced into effector cells. This kind of interpretation is not in line with the inhibition of cytotoxicity of NK cells in assays using mouse FcγR^+^ P815 cells. Indeed, if a murine antibody inhibits the lysis of P815 target cells, it means that the engagement of the receptor specifically recognized by that antibody delivers a negative signal on the activation of cytolysis. In this case, the antibody can transduce an inhibitory signal because the receptor is cross-linked by the antigen-binding fragment, and it is stabilized after the linking of the mAb Fc component with the FcγR^+^. This type of cytotoxic assay, called re-directed killing, has been applied in several reports to identify the activating or inhibiting function of some NK cell receptors [124,125,126]. Indeed, using anti-CD16 mAb or mAb to natural cytotoxicity receptors (NCRs) or other activating NK cell surface molecules, the NK cell-mediated killing of P815 cells can be activated [127,128,129,130,131]. On the contrary, mAbs to killer cell immunoglobulin-like receptor (KIR) or C-type lectin-like inhibitory receptor (CLIR), or other inhibitory receptors, block spontaneous or activating-receptor-induced P815 killing [127,128,129,130,131,132,133,134]. Furthermore, it has been reported that the NKRP1A/CD161 receptor was upregulated on NK cells by IL-12 [135,136] and that the CD16- or NKp46-mediated killing of P815 cells was inhibited by anti-NKRP1A/CD161-specific mAb [135]. This finding would further support that the engagement of NKRP1A/CD161 on NK cells may indeed deliver a negative signal. Overall, the role of NKRP1A/CD161 on cytotoxic cells in humans appears to be of an inhibitory type, and to date there are no reports indicating the presence of either inhibiting or activating isoforms, as demonstrated in rodent NK cells [48,49].

### 5.2. Role of NKRP1A/CD161 on Cell Precursors and T Cells

The effects of anti-NKRP1A/CD161 mAb on immature thymocytes, hematopoietic stem cell precursors (HSCs) and CD3^+^ T cells would indicate that the signal delivered through the engagement of this receptor is of the activating type [43,137,138]. Indeed, it has been shown that NKRP1A/CD161 can be expressed on a fraction of CD2^−^CD3^−^ co-expressing, at least in part, CD1a or CD34 or stem-cell-factor receptors. Both CD34^+^ and CD34^−^ thymocytes cultured with IL-2 can upregulate the NKRP1A/CD161 molecule at the cell surface. The anti-NKRP1A/CD161 mAb 191B8 can trigger proliferation of CD2^−^CD3^−^ immature thymocytes at similar degrees to those triggered by phytohemoagglutinin A (PHA), and the proliferative effect was not increased by the addition of exogenous IL-2. The phenotype of these immature thymocytes remained similar to that of the starting population, and no maturation to CD3^+^TCR^+^ T cells was detected [43]. About the role of NKRP1A/CD161 on immature NK cells, it has been shown that this molecule is among the earliest markers expressed during NK cell maturation from CD34^+^ HSP [137]. Indeed, in the initial stages of NK cell development, NKRP1A/CD161 is involved in the crosstalk of NK cell precursors with bone marrow stroma cells. The engagement of NKRP1A/CD161 with specific biotin-conjugated mAb followed by anti-biotin microbeads can induce the release of CXCL8 (also named IL-8). This is a potent chemokine that stimulates the chemotaxis/homing and phagocytic activity of granulocytes—neutrophils—during their migration to infection sites [137,138,139,140,141]. The release of CXCL8 was also triggered through NKp44 NCR cross-linking. However, NKRP1A/CD161 engagement did not trigger cytolytic activity or CD107a expression as a marker of degranulation, although NKp44 can also stimulate cytolysis of immature and mature NK cells. In addition, the crosslinking of NKRP1A/CD161 activates the PI3K-AKT pathway in addition to ERK1 and ERK2 [137]. This finding would suggest that cross-linking of NKRP1A/CD161 may be involved in localization of myeloid cells within tissues. Also, CXCL8 can have pro-angiogenic effects [138,139,140,141]. This property, associated with the chemotaxis on myeloid cells, can favor the generation of myeloid-derived suppressor cells and neo-angiogenesis within tumors. This would be interpreted as a negative event in the context of anti-tumor effects of NK cells. On the other hand, the functional role of the NKRP1A/CD161 molecule on Vα24 CD4^−^CD8^−^-invariant T cells proved that this molecule acts as a co-stimulatory molecule for CD1d recognition [142]. Importantly, the ligation of NKRP1A/CD161 did not trigger cytokine secretion such as IFN-γ, as happened for NK1^+^ murine T cells. In these experiments, it was demonstrated that plastic-coated anti-CD161 mAb, such as 191B8 or DX1, can markedly increase anti-CD3 mAb-mediated proliferation of some Vα24-invariant T cell clones [142]. The engagement of CD161 delivered a co-stimulatory signal more potent than that mediated by CD28 [142]. CD28 is the classical surface receptor, providing the second signal in conventional CD3^+^ T cells needed for the best T cell proliferation [143]. Furthermore, the engagement of CD94 with a specific mAb led to strong co-stimulation with anti-CD3 mAb. Importantly, the co-stimulation triggered by NKRP1A/CD161 and anti-CD3 mAb led to a strong increment of secreted IFN-γ and IL-4. However, these two cytokines were not released in the absence of cross-linking of CD3 receptors. Much more relevant, it was found that secretion of the cytokines IFN-γ and IL-4 by Vα24-invariant T cell clones upon interaction with CD1d C1R transfectants was inhibited by adding to the assay of the anti-NKRP1A/CD161 mAb. This effect was clear using the three different anti-NKRP1A/CD161 mAbs DX1, 191B8 and HP-3G10. Finally, the killing of CD1d^+^ C1R cells was not dependent on NKRP1A/CD161 surface receptor engagement, and this engagement did not increase the inhibitory effect mediated by anti-CD1d mAb. Overall, the co-stimulation of iNKT cells through CD1d and NKRP1A/CD161 involves a different molecular mechanism from those responsible for the activation of rodent Vα24-invariant T cells in which the NKRP1C is directly stimulatory, and this is associated with the p56^lck^ signal transducer [144,145]. A co-stimulatory effect of NKRP1A/CD161 and CD3 engagement was reported to lead to the optimal expansion of several peripheral blood lymphocytes and dendritic cells [145]. The anti-NKRP1A/CD161 mAb 191B8 and anti-CD3 mAb UCHT-1 were coated onto plastic on which PBMC were cultured and supplemented with high IL-2 (1000IU/mL) and granulocyte colony-stimulating factor (G-CSF 0–1013IU/mL). This mixture led to a strong expansion (more than 1000-fold) of both CD4, CD8 T cells and NKT cells expressing CD56 antigen. These cells exerted a strong anti-tumor effect against some tumor target cells. Based on these findings, it has been suggested that the use of this system could be best to generate cells to be administered for adoptive cell transfer [146]. However, it appears that expansion with the use of only anti-CD3 mAb and the best concentration of G-CSF are not shown in this report, hampering the correct interpretation of the data shown [147].However, it has been reported that the engagement of CD161 and CD3 in CD8^+^ T cells can inhibit TNF-α production [101], IFN-γ and TNF-α secretion in MAIT cells [147], and IFN-γ in promyelocytic leukemia zinc finger (PLZF)^+^ T cells [148]. These results, which conflict with those previously mentioned, could be explained by looking at the experimental system used, in addition to the differences in the effector cells analyzed. In the first case, the NKRP1A/CD161 receptor was engaged with P815 FcγR^+^ cells, which has likewise been reported with NK cells. The activation of CD8^+^ T cell triggering was inhibited but only for TNF-α release and not for CD3-mediated cytotoxicity. However, the molecular mechanisms underlining this inhibition were not investigated. The molecular second messengers involved are still to be defined. In the case of MAIT cells, NKRP1A/CD161 molecule engagement was achieved with soluble antibody on cells co-stimulated with different doses of anti-CD3 mAb and a fixed dose of anti-CD28 mAb linked to plastic. Finally, it should be noted that for PLZF^+^ T cells, the engagement of CD3 and NKRP1A/CD161 was performed for the first antibody as linked to plastic, while NKRP1A/CD161 was crosslinked with anti-biotin antibody after preincubation with biotin-labelled anti-NKRP1A/CD161 mAb. In summary, none of the reports analyzed in this chapter used the same methods to achieve engagement of NKRP1A/CD161. This is a strong limitation that does not allow us to make a definitive statement on the role of NKRP1A/CD161 on T cells. Furthermore, the inactivation of the NKRP1A/CD161 molecule could enhance the T-cell-mediated killing of glioma cells, leading to control of tumor growth [99,100]. In this case, the *KLRB1* gene was inactivated by gene editing, and the growth of two different humanized glioma models was delayed when these *KLRB1*-edited T cells were injected. In addition, the release of IFN-γ and IL-2 by T cells in a co-culture system of T cells and CLEC2D^+^ glioma cells was increased using an anti-NKRP1A/CD161 mAb that impaired the binding between the Fc human Ig chimeric molecule of CLEC2D and NKRP1A/CD161 on transfected Jurkat T cells. These last findings would suggest that CLEC2D expressed on glioma cells can deliver a negative signal through NKRP1A/CD161 in T cells. However, the direct demonstration that this interaction is effective in these experimental systems is not provided for either Jurkat T cell or T-glioma cell interaction. Furthermore, the putative inhibitory molecular mechanism elicited by the engagement of NKRP1A/CD161 during the interaction with CLEC2D^+^ glioma cells is not elucidated [99,100]. Overall, it is clear that the ligation of NKRP1A/CD161 can give a signal that affects some functional activities of NK and T cells, but a deeper analysis of the biochemical pathways involved is necessary to better understand the role of this molecule in immune cell activation and regulation. In the next chapter, we report some of the experimental evidence that can shed some insight into this specific topic.

## 6. NKRP1A/CD161-Mediated Signal Transduction

Downstream signaling through a receptor can involve tyrosine kinase activity, which can open calcium ion pores and lead to an increase in intracellular Ca^++^ concentration and activation of the production of calcium-dependent proteins and phosphoinositides, as well as of other second messengers, leading to the involvement of signaling enzymatic cascades [149,150]. These biochemical events can trigger the synthesis of cytokines, the release of granule content, and more complex events such as proliferation [148,151,152,153].

### NKRP1A/CD161-Mediated Activation of Enzymatic Cellular Components

**Figure 1:**
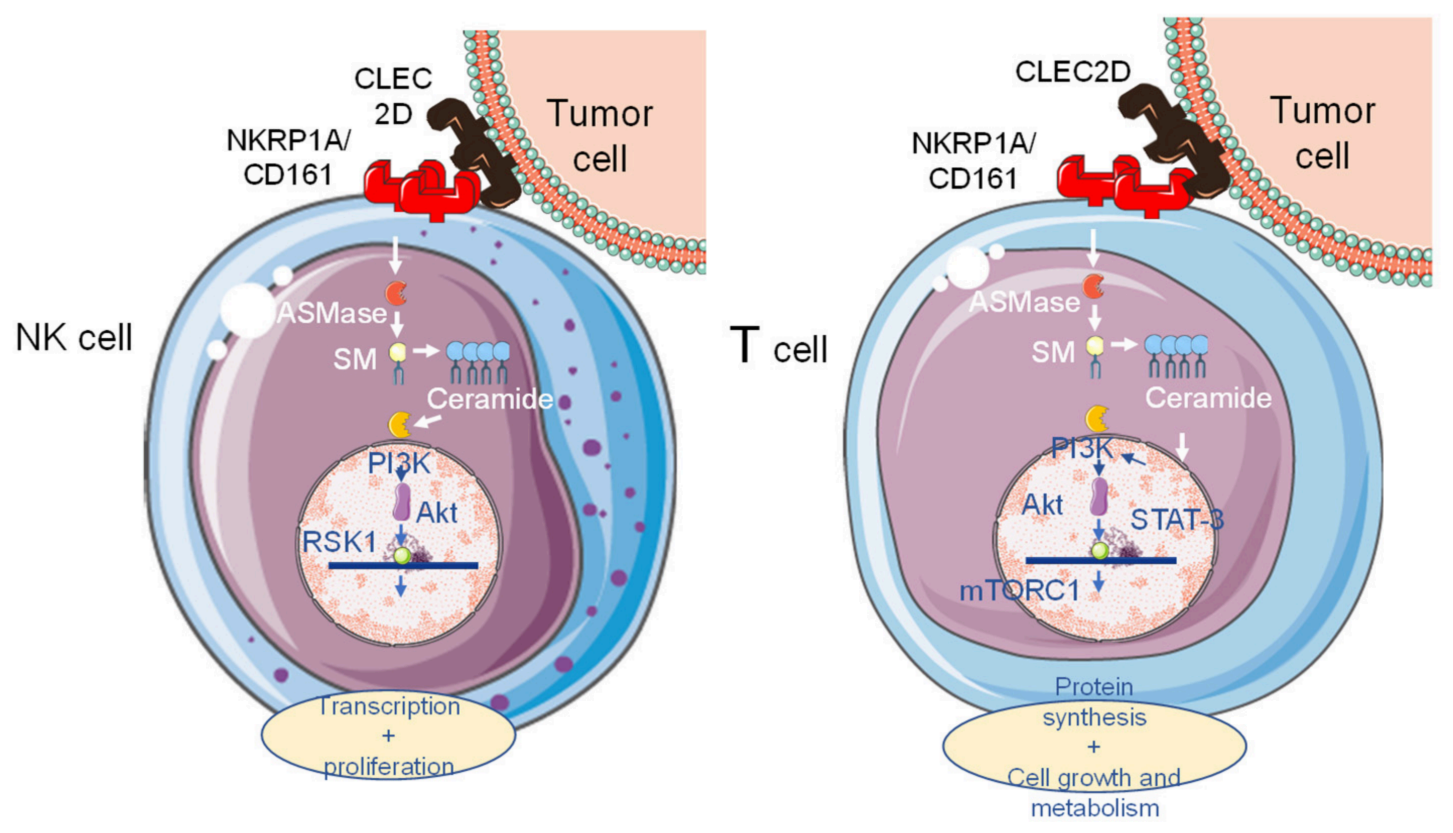
Putative association of NKRP1A/CD161 with acid sphingomyelinase (ASM). The engagement of NKRP1A/CD161 receptor on immune cells with the natural ligand CLEC2D present on tumor cells leads to the association and activation of acid sphingomyelinase (ASM). In turn, ASM leads to the generation of ceramide and consequent triggering of PI3K/AKT pathway involving, consequently, RSK1 and/or mTOR signal transduction. This may result in the production of cytokines or proliferation. The net effect will be dependent on the target cell involved in the interaction between NKRP1A/CD161 and CLEC2D. Distinct levels of ceramide and kinetics in the production of ceramide can influence the result of the receptor–ligand interaction. Usually, the effect on NK cells leads to inhibition of NK-cell-specific activities, while T cells can determine a strong co-stimulation of CD3/T-cell-receptor-mediated proliferation. The arrows show the sequence of the events that follow the ligand-receptor interactions. The figure was produced using templates from the Anatomy and Human Body and Cellular Biology series of Smart Servier Medical Art https://smart.servier.com/ (accessed on 10 August 2026) with some modifications.

The NKRP1A/CD161 receptor does not contain ITAM or ITIM domains, which can suggest a signal transduction involving the direct activation or inhibition of tyrosine kinase [22,154,155]. To identify proteins involved in binding with the cytosolic domain of human NKRP1A/CD161, the yeast two-hybrid system was used to screen candidates [41]. Acidic sphingomyelinase (ASM) was identified by this screening (Figure 1).

This association was confirmed by immunoprecipitation experiments with digitonin after cross-linking with anti-NKRP1A/CD161 mAb. The enzymatic activity of ASM was increased after the engagement of NKRP1A/CD161 with the generation of ceramide from sphingolipid catabolism. Importantly, ceramide can influence the activation of protein kinase C, protein phosphatases PP1 and PP2A, and ceramide-activated kinase/kinase suppressor of Ras [156,157]. In turn, these enzymes can lead to altered phosphorylation of Akt/PKB and proline-directed kinases such as MAPK [158]. The ligation of NKRP1A/CD161 induced a three-fold activation of PKB/Akt and Rsk1 kinases in the NK cell line YT transfected with NKRP1A/CD161. This effect was reduced by the wortmannin inhibitor of PI3K and by pretreatment with SR33557, an inhibitor of ASM [41]. The proliferation of NK T cells triggered with cross-linking of NKRP1A/CD161 with suboptimal doses of anti-CD3 mAb was inhibited in the presence of the ASM inhibitors SR33557 or imipramine [41]. Similarly, the increase in IFN-γ release in NK T cells determined with the co-engagement of CD3 and NKRP1A/CD161 molecules was strongly inhibited with imipramine [41]. Importantly, CD161-associated ASM activity was found only in the membrane raft fractions. Overall, these findings indicate that NKRP1A/CD161 engagement generates ceramide, which triggers signals associated with increased survival, in agreement with the activation of PKB/Akt [159]. This behavior is like other classical co-stimulatory molecules like CD28 and LFA-1 [160,161].

Along this line, it has been reported that ASM can also have important functional significance in CD4^+^ αβT cells [162,163]. In this regard, it has been shown that NKRP1A/CD161 can interact with ASM on Th17 cells, inducing an intracellular cascade activating STAT3, which is involved in cell survival and proliferation and mTOR pathways, regulators of cell growth and metabolism [162,163]. It is of note that the cross-linking of NKRP1A/CD161 and CD39 led to an increase in ceramide CD39-mediated production in CD39^+^CD161^+^ CD4^+^ T cells, which typically exhibit a molecular signature of Th17 cells [162]. This finding confirmed the key role of ASM and ceramide in NKRP1A/CD161 signaling. Furthermore, it has been reported in the subset of Vδ2 γδT cells that the engagement of NKRP1A/CD161 can trigger the activation of CAMKII [44]. Pharmacological inhibition of CAMKII with KN62 and KN93, but not with the inactive compound KN92, blocked this activation, suggesting that this targeting may influence NKRP1A/CD161 signals. Overall, the NKRP1A/CD161 signaling pathway may involve different enzymatic activities, and the final effect could be related to the functional properties of the specific cell subsets expressing NKRP1A/CD161. The co-engagement of other functional receptors may lead to either triggering or inhibition depending on the repertoire of these receptors on the immune cell of interest.

## 7. NKRP1A/CD161 Involvement in Trans-Endothelial Migration

**Figure 2:**
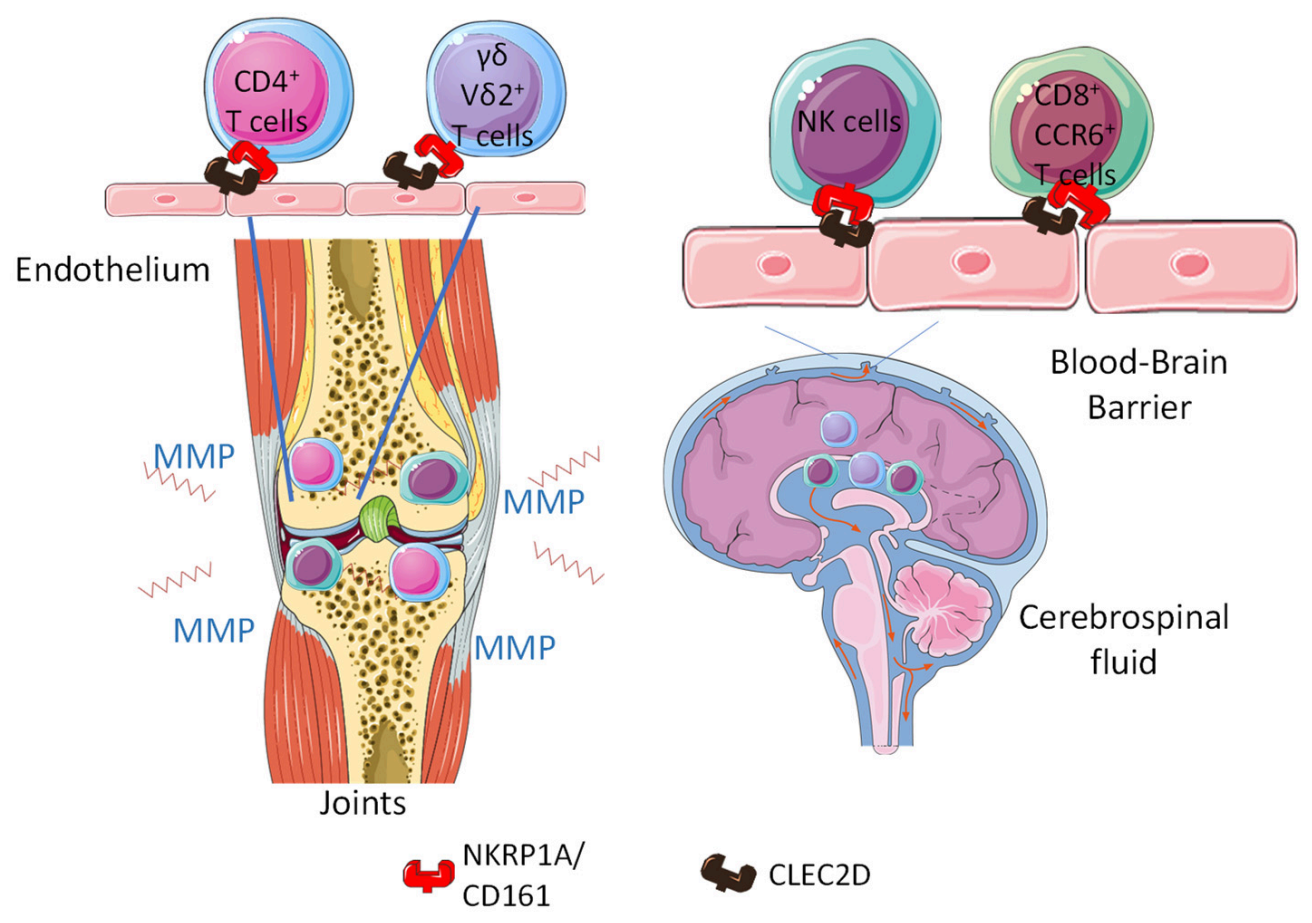
NKRP1A/CD161 may be involved in transendothelial migration. Immune cells expressing NKRP1A/CD161 can migrate through endothelial cells to localize in specific inflammed tissues such as the joint in rheumatoid arthritis (RA) or the central nervous system in cerebrospinal fluid (CSF) in multiple sclerosis (MS). The cells may localize and upregulate the NKRP1A/CD161 receptor upon stimulation with cytokines such as IL-12 (see the text), while the CLEC2D molecule may be putatively upregulated on endothelial cells and in the epithelial and stromal components of inflammed tissue. The arrows show where immune cells can be localized migrating into the cerebrospinal fluid. Figures were designed from the templates of the Anatomy and Human Body and Cellular Biology series of Smart Servier Medical Art https://smart.servier.com/ (accessed on 10 August 2026).

The tissue localization of effector or regulatory immune cells during infections, inflammation, or neoplastic cell growth is a key step to trigger the host immune response to different noxae [164,165,166,167]. The process of leukocyte homing is complex and involves some key molecules such as selectins, integrins such as LFA1 and its counter-receptor intercellular adhesion molecule (ICAM) 1, vascular endothelial (VE)–cadherin and platelet endothelial cell molecule (PECAM)1/CD31), and the single-chain type-1 glycoprotein MIC2/CD99 [166,167]. There is some evidence in the literature that suggests the role of NKRP1A/CD161 as one of the molecules involved in trans-endothelial migration (44,168,169). Firstly, it has been shown that anti-NKRP1A/CD161 mAb can interfere, in an in vitro system, with the transmigration of peripheral blood NKRP1A/CD161^+^CD4^+^ T cells through umbilical endothelial cell monolayers [168]. This CD4^+^ T cell subset co-expressed markers of memory cells and migrate quicker compared to the NKRP1A/CD161^−^CD4^+^ counterpart [168]. A similar role of NKRP1A/CD161 in transmigration has been reported for γδ Vδ2^+^ T cells both in healthy donors (HDs) and multiple sclerosis (MS) patients [44,169]. Again, antibodies to NKRP1A/CD161 can inhibit endothelial transmigration in Vδ2+ T cell clones, and this inhibition was also evident in Vδ2^+^ T cells that had been upregulated in the NKRP1A/CD161 upon exposure to IL-12. Overall, the inhibition of migratory capabilities can be increased using a combination of anti-LFA1 and anti-NKRP1A/CD161 mAbs. It is of note that an increase in γδ T cell NKRP1A/CD161^high^ CCR6^+^ has been found in cerebrospinal fluid (CSF) of MS patients but not during infectious diseases or in healthy donors [169,170,171]. Furthermore, NKRP1A/CD161^high^CD8^+^ CCR6^+^ were increased in MS patients and present in intralesional immune infiltrates [163], and they can secrete IFN-γ. Altogether, these findings would suggest that the presence of NKRP1A/CD161 can identify a lymphocyte population with potential pathogenic properties in MS. It is still to be figured out whether NKRP1A/CD161 serves as a receptor to localize γδT cells into the brain, or whether it can function to favor recirculation from the brain to the peripheral blood. The possible involvement of NKRP1A/CD161 in tissue homing of subset immune cells has been reported for CD4^+^ T cells in rheumatoid arthritis (RA) and mucosal-associated invariant T cells (MAITs) in MS and amyotrophic lateral sclerosis (ALS) [172,173]. In detail, a decrease in the level of NKRP1A/CD161 expression has been found in peripheral blood during progressive MS and ALS, suggesting a redistribution of CD8^+^ MAITs [172]. The CD3^+^ TCR γδ^−^ Vα7.2^+^NKRP1A/CD161^high^ MAIT cells’ presence correlated with an MS clinical and radiological intra-lesion course. Furthermore, the MAIT cell migration through the blood–brain barrier was supported by their increase in CSF than in peripheral blood. On the other hand, CD4^+^CD161^+^ T cells can be considered a predictive marker of disease activity, as they increased both in the peripheral blood and synovial fluid (SF) of RA patients [173,174,175] Furthermore, CD98 and CD147, surface receptors involved in the recruitment of inflammatory cells into the SF of RA patients and progression of the disease, were present on CD4^+^CD161^+^ T cells. This suggests that NKRP1A/CD161-expressing cells display the property of producing metalloproteinases (MMP) through CD147 and trigger metabolic properties via CD98 to favor localization in the SF and the increase of inflammation in RA patients [173,176]. All these findings support that cells expressing the NKRP1A/CD161 receptor can actively transmigrate from the peripheral blood to the tissues and vice versa. This would suggest that the targeting of this molecule can provide an useful mean to regulate the localization of different effector or regulating cells (Figure 2).

### Role of NKRP1A/CD161 in the TME

**Figure 3:**
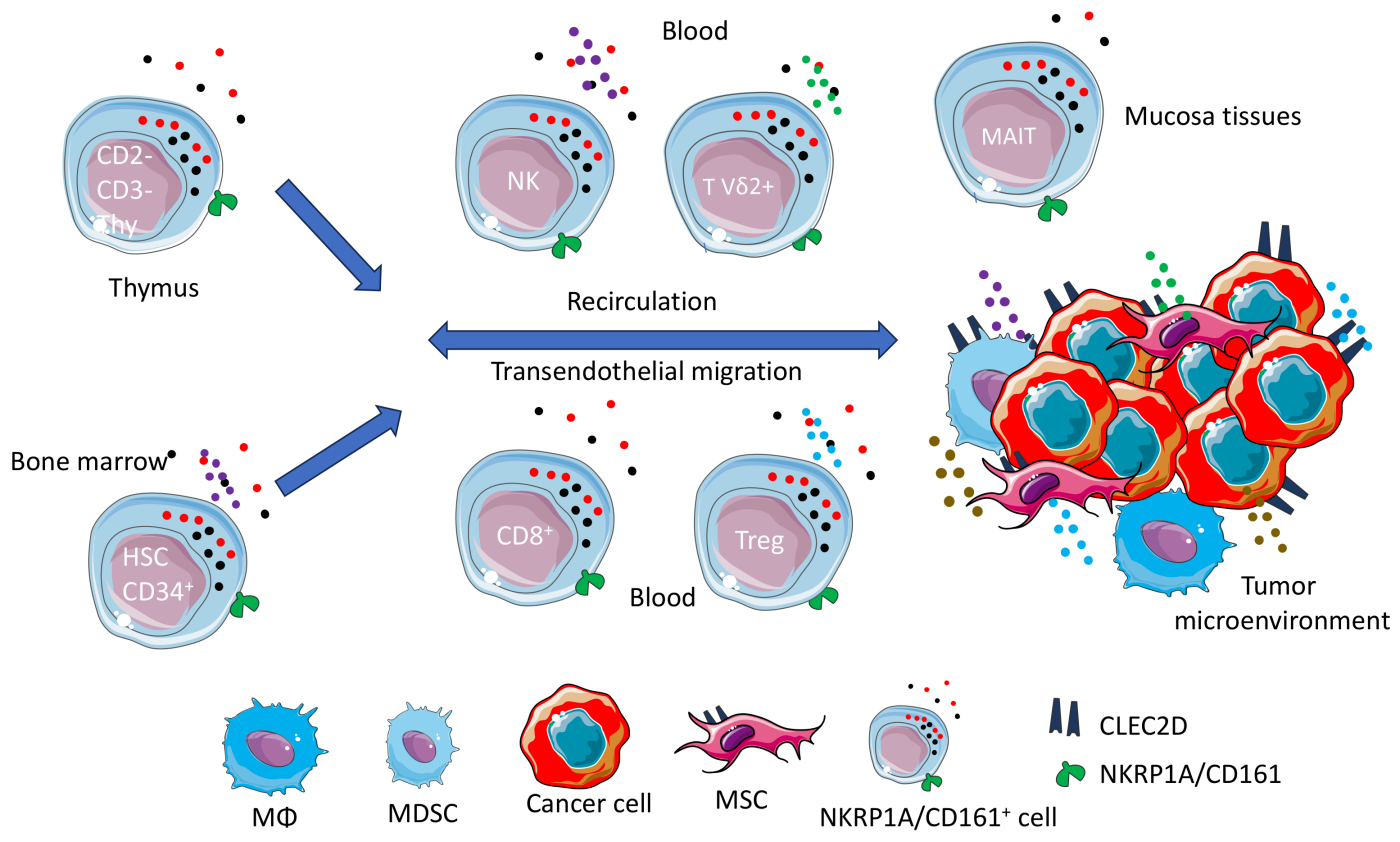
Possible roles of NKRP1A/CD161 immune cells in the tumor microenvironment (TME). Different types of immune cells can express NKRP1A/CD161 at the cell surface, such as NK cells, CD4^+^T cells, CD8^+^ T cell, Vδ2+ γδT cells, and invariant mucosal-associated T cells (MAITs), in addition to hematopoietic stem cell precursors (HSCs) or CD2^−^CD3^−^-immature thymocytes. These cells can migrate from specific tissue into the blood and/or localize into the TME. Although not shown, in this cartoon, the increase in NKRP1A/CD161^+^CD8^+^ T cells within the tumor mass of esophageal squamous cell carcinoma can favor the anti-tumor immune response. Alternatively, the blocking of the interaction of NKRP1A/CD161 with CLEC2D in glioblastoma can improve the anti-tumor immune response. The immune cells may recirculate from the tumor mass to the peripheral blood and from the periphery to other tissues. It is conceivable that the role of NKRP1A/CD161 receptor is dependent on the specific features of immune cells expressing this receptor. The arrows show the migration of cells from the primary lymphoid tissues into the peripheral blood and their localization into mucosal tissues and recirculation. The figures were designed from the templates of the Anatomy and Human Body and Cellular Biology series of Smart Servier Medical Art https://smart.servier.com/ (accessed on 10 August 2026).

The role of the TME in shaping the growth of tumor cells and anti-tumor immune response is well established [177,178,179,180,181,182]. The contribution of NKRP1A/CD161’s interaction with CLEC2D in the TME was reviewed recently [114]. In detail, this interaction modulates the immune response, but the lack of a specific functional homologous molecule in the rodent model has impaired the exact definition of the role of this molecule. However, the studies and bioinformatic analysis have added some new insights into cancer [114,182,183,184,185,186,187]. In the previous chapters, some of the reports regarding the expression and putative function of NKRP1A/CD161-CLEC2D interactions in different types of cancers were considered in a certain detail. Herein, we focus on some paradigmatic examples that highlight the role of this crosstalk between effector cells and tumor cells. Study of the clinical significance of NKRP1A/CD161^+^CD8^+^ T cells in esophageal squamous cell carcinoma (ESCC) has been performed by applying single-cell sequencing and modelling computer tomography (CT)-based imaging to evaluate the infiltration [186]. The approach consisted of several steps, comprising: a) analysis of a large clinical cohort from different centers with prognostic validation to different therapeutic treatments; b) the prediction of therapeutic response considering NKRP1A/CD161^+^CD8^+^ T cells and response to anti-PDL1 antibody; c) single-cell sequencing to identify the major molecular mechanisms involved using cell communication and functional enrichment analyses; d) analysis of systemic immunity to contextualize this aspect with the TME composition; e) modelling based on clinical imaging and segmentation, then generating a model and validating it [186]. Based on these analyses, it has been shown that the TIL NKRP1A/CD161^+^CD8^+^ can serve as a prognostic biomarker, predictive of the efficiency of therapy, and associated with therapeutic response. The single-cell RNA sequence (scRNA-seq) in paired samples of neoadjuvant immunotherapy combined with the chemotherapy (NICT) cohort with major pathological response (MPR) versus non MPR patients revealed that responder patients were particularly enriched with TIL NKRP1A/CD161^+^CD8^+^ cells. This cell subset was characterized by functional pathways involved in T cell triggering, proliferation, cytotoxicity, and anti-tumor effector processes, as assessed by gene set enrichment analysis (GSEA). This feature was associated with macrophages expressing the co-stimulatory TNFSF9 (4-1BBL) while expressed the counter receptor TNFRSF9 (4-1BB) [186]. It is of note that in MPR patients, a Th1 response in terms of cytokines present in peripheral blood was present after immunotherapy treatment. However, this response was not detected in non MPR patients, lacking the NKRP1A/CD161^+^CD8^+^ cells. These findings would support the production of IFN-γ and TNF-α together with co-stimulation, leading to a self-reinforcing positive loop between T and macrophages in the tumor site. This loop can handle the local anti-tumor immune response along with sustained co-stimulatory signaling, improving the immune response efficacy and survival. On the other hand, the MAIT cells CD3^+^NKRP1A/CD161^+^Vα7.2 present in peripheral blood and within the liver in hepatocellular carcinomas (HCCs) produced less IFN-γ after stimulation with cytokines such as IL-12/IL-18 [183]. The scRNA-seq of this cell population from HCC indicates that these cells showed some features of exhausted lymphocytes and a strong upregulation of the inhibitory checkpoint LAG-3 [183]. Altogether, these findings would indicate that the correct identification of the role of NKRP1A/CD161 in the TME is still controversial. Figure 3 shows a putative scenario of the TME and the involvement of NKRP1A/CD161.

## 8. Therapeutic Perspectives and Future Directions

### 8.1. NKRP1A as a Target for Tumor Immunotherapy

The growing understanding of the NKRP1A/CD161–CLEC2D pair as a non-conventional regulatory axis has paved the way for targeted pharmacological strategies but has also highlighted specific risks related to its still undefined precise role. In oncology, the therapeutic rationale is twofold: on the one hand, inhibition of NKRP1A/CD161 signaling on TIL could “release” effector functions blocked in the neoplastic microenvironment; on the other, blockade of CLEC2D on tumor and stromal cells could restore NK and T cell-mediated cytotoxicity and remove an upstream immune brake [99,100,114]. Functional and transcriptomic data have indeed shown that NKRP1A/CD161 is upregulated in TILs of many solid tumors and that CLEC2D is expressed by both tumor cells and infiltrating myeloid compartments, strengthening the idea of a “network” checkpoint involving multiple arms of anti-tumor immunity [22,114]. In preclinical models, anti-CD161 antibody increased cytotoxicity, cytokine production, and TIL proliferation, suggesting potential synergy with checkpoint inhibitors (anti-PD1/PDL1). These observations have recently been extended to hematological malignancies, where NKRP1A targeting improves T cell function against B-derived cell lines, broadening the spectrum of potential indications [120]. In parallel, the CLEC2D axis has emerged as a tumor-intrinsic target. Analyses of TCGA cohorts and clinical datasets indicate that elevated levels of *LLT1* are associated with immunosuppressive microenvironments and NK evasion phenotypes; CLEC2D blockade is therefore proposed as a strategy to “reignite” immunosurveillance, particularly in tumors poorly responsive to ICI [118]. On the clinical front, first experiences have emerged: ZM008 (anti-CLEC2D antibody) has shown preliminary signals of biological activity and feasibility in early settings [118], while IMT-009 (anti-NKRP1A/CD161 antibody) is under development in phase 1/2a studies on solid tumors and hematological malignancies, supported by preclinical evidence of restored T and NK cytotoxicity [188]. These programs—still at an early stage—will be crucial to define the therapeutic window, selection of biomarkers (primarily NKRP1A/CD161 or CLEC2D co-expression), and optimal combinations with ICI or cellular therapies [118,188,189,190].

### 8.2. Limitation of NKRP1A-Directed Therapy

The biological context remains decisive for clinical success. Experience in gliomas has already provided proof of the principle of the axis “druggability,” showing that NKRP1A/CD161 blockade can reverse a functional brake in TILs and enhance anti-tumor activity; however, studies on tumor-specific CD4^+^ T cells have also documented that, in certain settings, NKRP1A/CD161 does not act as a true inhibitory checkpoint but rather as a marker of “rapid responder” populations activatable by therapeutic vaccination. The coexistence of these outcomes highlights the urgency of fine patient stratification, integrating NKRP1A/CD161-CLEC2D levels and heterogeneity, immune composition of the TME, and the functional state of TILs, to avoid “one-size-fits-all” interventions [95,99,100,101,120]. Alongside opportunities, the systemic NKRP1A/CD161-CLEC2D blockade raises safety issues. The receptor is linked to the Th17/ “type-17” compartment, and to the biology of MAIT and NK cells; its modulation may thus interfere with the balance between antimicrobial defense and inflammation [62,114]. In chronic infection scenarios, NKRP1A/CD161^+^ T and MAIT cells contribute to early pathogen control, but persistent stimulation can lead to dysfunction; at the same time, in autoimmunity the NKRP1A–STAT3/mTOR circuit is associated with IL-17 production and inflammatory persistence. The practical consequence is that indiscriminate removal of the NKRP1A/CD161-imposed brake could increase the risk of inflammatory events or, conversely, susceptibility to infections, especially if combined with other ICIs. This trade-off requires targeted monitoring (microbiome, “type-17” transcriptomic signatures) and gradual combination designs, prioritizing settings with a high benefit–risk ratio and patients with clear LLT1 axis dominance in the TME [114,120,122].

On the mechanistic level, further insights come from downstream NKRP1A/CD161 is signaling. Activation of acid sphingomyelinase and ceramide generation provides a possible metabolic hub that could modulate the outcome of signaling (inhibitory or co-stimulatory) depending on the cellular state and cytokine milieu. This opens exploratory hypotheses for combinations with agents that reshape sphingolipid metabolism or with STAT3/mTOR modulators, to “steer” functional polarity in specific T or NK populations. Systematic studies, however, are needed to avoid undesirable interference with physiological antimicrobial responses and Th17 plasticity [190,191,192,193].

### 8.3. Future Perspectives

In the short-to-medium term, three directions are priorities. First is the development of companion assays that standardize measurement of NKRP1A/CD161-CLEC2D expression on effector cells and tumor cells. It is relevant to characterize the CLEC2D protein or LLT1 mRNA subcellular and histotopographic localization in the TME, to guide enrollment and predict response; ongoing clinical programs are already incorporating biomarker-driven selection criteria, including dual target–ligand positivity. Second is the definition of rational combinations: anti-NKRP1A/CD161 and anti-CLEC2D mAb with anti-PDL1, with IL-12/IL-18 agonists, or with cell therapies (e.g., CAR-T) could maximize effector reactivation but must be balanced against the risk of systemic inflammation. Third, extension beyond oncology: in autoimmunity and transplantation, selective modulation (not necessarily blockade) of the axis could be hypothesized to disarm pathogenic Th17 circuits or reduce rejection, while preserving antimicrobial surveillance functions. The key, once again, will be the use of contextual therapeutic schemes and mechanistic biomarkers, not the simple “on/off” dichotomy of the receptor. In summary, NKRP1A/CD161 confirms itself as a “precision” rather than a “universal” target: promising where the NKRP1A/CD161–CLEC2D axis dominates tumor-intrinsic and TIL-intrinsic immune resistance, which is potentially risky where its modulation disrupts Th17/MAIT circuits crucial for mucosal homeostasis and pathogen defense. The maturation of clinical data on anti-NKRP1A/CD161 (IMT-009) and anti-CLEC2D (ZM008), together with an armamentarium of integrated biomarkers, will tell whether this promise can translate into concrete and sustainable clinical benefits.

Ultimately, NKRP1A/CD161 emerges not as a simple inhibitory or co-stimulatory receptor, but as a non-conventional immune checkpoint whose modulation requires a precision approach—capable of harnessing its therapeutic potential without compromising the physiological balance between protective immunity and tolerance.

## 9. Conclusions

Based on the reported findings and discussion, the NKRP1A/CD161 surface receptor may play opposing roles identified with different procedures and reagents. The generation of other and well-characterized anti-NKRP1A/CD161 reagents could provide further relevant information. The use of the present NKRP1A/CD161 reagents in either preclinical models or clinical trials should consider that in the TME, NKRP1A/CD161^+^ cells with opposite functions are present. Furthermore, the heterogeneity of the TME in different portions of each tumor and the presence of soluble or exosome-associated NKRP1A/CD161 ligands such as CLEC2D may greatly influence the patient’s response. Nowadays, the availability of a wide range of potent and specific targetable drugs triggers researchers and clinicians to test them soon. This habit should be avoided for target molecules with an incomplete definition of their features, such as NKRP1A/CD161.

## Figures and Tables

**Table 1:** Expression and function of NKRP1A and NKRP1 family members in humans and rodents.

Name of the Molecule	Gene Coding for the Protein and Location	Expression and Some Aliases	Functional Role and Similar Molecules	Identified Ligands	Main Ref.
Killer cell lectin-like receptor subfamily B member 1	Chromosome 12 *KLRB1*/12p13.31	Human NK, NKT, MAIT, αβT, γδT, iNKT Vα24, γδT monocyte, DC, TIL Aliases CD161, CLEC5B, NKR, NKR-P1, NKR-P1A, NKRP1A, hNKR-P1A, killer cell lectin-like receptor B1	Inhibiting co-stimulatory adhesion molecule	CLEC2D protein codified by *LLT1* gene	[49,57,72]
Killer cell lectin-like receptor subfamily B member 1A	Chromosome 6 klrb1/klrb1a 6 F3|6 63.06 cM	Mouse NK cells and innate-like T cells Aliases Ly55a, NKR-P1.7, NKR-P1A, NKRP12, Nkrp1-a, Nkrp1a, ly-55A, killer cell lectin-like receptor subfamily B member 1A	NKRP1A/ NKRP1F activating NKRP1B inhibiting NK1.1; NKRP1C activating NKRP1G Nkrp1g inhibiting	Clr-b for NKRP1B and NKRP1D Clr-c, d, g for NKRP1F but not with Clr-11 or Clr-b Clr-d,f,g for NKRP1G but not with Clr-11 or Clr-b	[42,56,73,74,75,76,77]
Killer cell lectin-like receptor subfamily B member 1	Chromosome 4 nkrp1a & nkrp1b Located in the centromeric cluster	Rat NK cells and innate-like T cells	Nkrp1a activating Nkrp1b inhibiting Nkrp1f activating (mainly) Nkrp1b recognition RCMV *	Clr-11 for NKRP1A and NKRP1B Clr-2,3,4,6,7, for NKRP1F Clr-2,4,6,7 for NKRP1G C-type lectin-like (RCTL) gene product Homologous to Clr for NKRP1B *	[42,73,74,75]

NK: natural killer; NK-T: NK like T cells; MAIT: mucosal-associated invariant T; DC: dendritic cells; TIL: tumor infiltrating lymphocytes; CLEC2D: C-type lectin domain family 2 member D; *LLT1*: lectin-like transcript-1; NKRP1: natural killer cell receptor protein 1; RCMV: rat cytomegalovirus; * molecules involved in recognition of RCMV besides the Ly49 rat system. Some relevant references and further details are [78,79,80] (mouse), [81,82,83] (rat), ref. [84] (overview of rodents and humans).

**Table 2:** The cellular expression of CLEC2D: the ligand of NKRP1A/CD161 receptor.

Type of Tissue Cells	Immune Cell Basal Expression	Immune Cells Upon Activation	Other Healthy Cells	Tumor Cells
Human	Resting B cells Germinal center cells Plasmablasts Very low expression on resting lymphocytes	Strong induction by cytokines and upon activation TLR, TNF, IL-1β IFN-γ, PHA, anti-CD3mAb anti-CD16 mAb	Brain Endocrine system Proximal digestive tract Endothelial cells	Epithelial tumors Mesenchymal tumors Hematological tumors
Mouse	Plasmacytoid dendritic cells Subsets of T lymphocytes Leukocytes Dendritic cells and antigen-presenting cells		Non hematopoietic cells Liver, endothelium, brain microglia Kidney, testes, gastrointestinal tract	Widely expressed hematopoietic and non-hematopoietic cells
Rat	B cells, APC Leukocyte subsets: granulocytes, myeloid cells, lymphocytes, splenic lymphoid follicles, thymocytes		Skeletal muscle, Endothelial cells Intestine kidney, lung	Widely expressed hematopoietic and non-hematopoietic cells

TLR: toll-like receptor; TNF: tumor necrosis factor; IL-1β: interleukin 1β; IFN-γ: interferon γ; PHA: phytohemagglutinin A; APC: antigen-presenting cells.

## Data Availability

No new data were created or analyzed in this study. Data sharing is not applicable to this article.

## Abbreviations

The following abbreviations are used in this manuscript:

A2M	Alpha-2-macroglobulin
AICL	Activation-induced C-type lectin
AKT	Protein kinase B (AKT)
ALS	Amyotrophic lateral sclerosis
AML	Acute myeloid leukemia
APC	Antigen-presenting cell
ARG1	Arginase 1
ASM	Acid sphingomyelinase
BAD	Bcl-2-associated death promoter
BLCA	Bladder urothelial carcinoma
BRCA	Breast-invasive carcinoma
CAMKII	Ca^2+^/calmodulin-dependent kinase II
Ca^2+^	Calcium ion
CARD	Caspase activation and recruitment domain
CD161	Cluster of Differentiation 161
CESC	Cervical squamous cell carcinoma and endocervical adenocarcinoma
CHOL	Cholangiocarcinoma
CLEC4A	C-type lectin domain family 4 member A
CLEC5B	C-type lectin domain family 5 member B
CLIR	C-type lectin-like inhibitory receptor
CLL	Chronic lymphocytic leukemia
CLR	C-type lectin receptor
CML	Chronic myeloid leukemia
CMV	Cytomegalovirus
CNV	Copy-number variation
COAD	Colon adenocarcinoma
CRI	Cancer Research Institute
CSF	Cerebrospinal fluid
CT	Computed tomography
CTLA4	Cytotoxic T-lymphocyte-associated protein 4
CXCL8	C-X-C motif chemokine ligand 8
DC	Dendritic cell
DC-SIGN	Dendritic cell-specific intercellular adhesion molecule-3-grabbing non-integrin
EBV	Epstein–Barr virus
ENTPD1	Ectonucleoside triphosphate diphosphohydrolase 1
ERK	Extracellular signal-regulated kinase
ESCA	Esophageal carcinoma
Fc	Fragment crystallizable
FDC	Follicular dendritic cell
FOXM1	Forkhead box protein M1
FOXP3	Forkhead box protein P3
G-CSF	Granulocyte colony-stimulating factor
Gal-9	Galectin-9
GBM	Glioblastoma multiforme
GSEA	Gene set enrichment analysis
HCC	Hepatocellular carcinoma
HD	Healthy donor
HIV	Human immunodeficiency virus
HLA	Human leukocyte antigen
HNSC	Head and neck squamous cell carcinoma
hNKR-P1A	Human NKR-P1A
HPV	Human papillomavirus
HSPC	Hematopoietic stem and progenitor cell
ICAM	Intercellular adhesion molecule
ICI	Immune checkpoint inhibitor
ICRs	Immune-checkpoint receptors
ICOS	Inducible T-cell costimulator
ICR	Immune checkpoint receptor
IDH	Isocitrate dehydrogenase
IDO1	Indoleamine 2,3-dioxygenase 1
IFN-γ	Interferon-γ
Ig	Immunoglobulin
IL-17	Interleukin-17
iNKT	Invariant natural killer T cell
ITAM	Immunoreceptor tyrosine-based activation motif
ITIM	Immunoreceptor tyrosine-based inhibitory motif
KACL	Keratinocyte-associated C-type lectin
KICH	Kidney chromophobe carcinoma
KIR	Killer cell immunoglobulin-like receptor
KIRC	Kidney renal clear cell carcinoma
KIRP	Kidney renal papillary cell carcinoma
KLRB1	Killer cell lectin-like receptor subfamily B member 1
LAG3	Lymphocyte activation gene 3
LAMP1	Lysosomal-associated membrane protein 1
LFA-1	Lymphocyte function-associated antigen 1
LIHC	Liver hepatocellular carcinoma
LLT1	Lectin-like transcript 1
LKB1	Liver kinase B1
LPS	Lipopolysaccharide
LUAD	Lung adenocarcinoma
LUSC	Lung squamous cell carcinoma
mAb	Monoclonal antibody
MAIT	Mucosal-associated invariant T cell
MBL	mannose-binding lectin
MFI	Mean fluorescence intensity
MHC	Major histocompatibility complex
MIC2	Microneme protein 2
MMP	Matrix metalloproteinase
MPR	Major pathological response
mRNA	Messenger RNA
MS	Multiple sclerosis
MUM1	Multiple myeloma oncogene 1 (IRF4)
mTOR	Mammalian target of rapamycin
NCR	Natural cytotoxicity receptor
NICT	Neoadjuvant immunotherapy combined with chemotherapy
NK	Natural killer cell
NKC	Natural killer gene complex
NKRP1	Natural killer receptor protein 1
NKT	Natural killer T cell
NKR	Natural killer receptor
NLR	NOD-like receptor
NLRC4	NOD-like receptor family CARD domain-containing protein 4
NSCLC	Non-small cell lung cancer
OCIL	Osteoclast inhibitory lectin
OPSCC	Oropharyngeal squamous cell carcinoma
PAAD	Pancreatic adenocarcinoma
PCPG	Pheochromocytoma and paraganglioma
PD1	Programmed cell death protein 1
PDL1	Programmed death-ligand 1
pDC	Plasmacytoid dendritic cell
PECAM	Platelet endothelial cell adhesion molecule
PHA	Phytohemagglutinin A
PI3K	Phosphatidylinositol 3-kinase
PLZF	Promyelocytic leukemia zinc finger protein
PMA	Phorbol 12-myristate 13-acetate
PRAD	Prostate adenocarcinoma
PTEN	Phosphatase and tensin homolog
RA	Rheumatoid arthritis
RORγt (RORC)	RAR-related orphan receptor gamma t
SARC	Sarcoma
scRNA-seq	Single-cell RNA sequencing
SF	Synovial fluid
SKCM	Skin cutaneous melanoma
SLAMF9	Signaling lymphocytic activation molecule family member 9
SOX	SRY-related HMG-box transcription factor
STAD	Stomach adenocarcinoma
STAT1	Signal transducer and activator of transcription 1
TCGA	The Cancer Genome Atlas
TCR	T-cell receptor
TGF-β	Transforming growth factor-β
Th17	T helper 17 cell
THCA	Thyroid carcinoma
THYM	Thymoma
TIL	Tumor-infiltrating lymphocyte
Tim-3	T-cell immunoglobulin and mucin domain-containing protein 3
TLR4	Toll-like receptor 4
TME	Tumor microenvironment
TNF	Tumor necrosis factor
ULBP1	UL16-binding protein 1
VE	Vascular endothelium
αβ T cell	Alpha-beta T cell
